# Evaluation of Three Devices for the Isolation of the Stromal Vascular Fraction from Adipose Tissue and for ASC Culture: A Comparative Study

**DOI:** 10.1155/2017/9289213

**Published:** 2017-02-22

**Authors:** Jonathan Rodriguez, Anne-Sophie Pratta, Nacira Abbassi, Hugo Fabre, Fanny Rodriguez, Cyrille Debard, Jacqueline Adobati, Fabien Boucher, Frédéric Mallein-Gerin, Céline Auxenfans, Odile Damour, Ali Mojallal

**Affiliations:** ^1^Banque de Tissus et Cellules, Laboratoire des Substituts Cutanés, Hôpital Edouard Herriot, Hospices Civils de Lyon, 5 Place d'Arsonval, Pavillon I, 69437 Lyon, France; ^2^Lyon University, CarMeN Laboratory, INSERM U1060, 69008 Lyon, France; ^3^Laboratoire de Biologie Tissulaire et Ingénierie Thérapeutique, UMR CNRS 5305, Université Lyon 1, Lyon, France; ^4^Laboratory for Regenerative Technologies, Department of Biomedical Engineering, University of Basel, 4123 Allschwil, Switzerland; ^5^Laboratoire Central d'Anatomie Pathologique, Hôpital Édouard Herriot, Lyon, France; ^6^Department of Plastic, Reconstructive and Aesthetic Surgery, Croix Rousse Hospital, Hospices Civils de Lyon, University of Lyon, UCBL1, Lyon, France

## Abstract

Adipose-derived stem/stromal cells (ASCs) reside in the stromal vascular fraction (SVF) of adipose tissue (AT) and can be easily isolated. However, extraction of the SVF from lipoaspirate is a critical step in generating ASC, and semiautomated devices have been developed to enhance the efficacy and reproducibility of the outcomes and to decrease manipulation and contamination. In this study, we compared the reference method used in our lab for SVF isolation from lipoaspirate, with three medical devices: GID SVF-1™, Puregraft™, and Stem.pras®. Cell yield and their viability were evaluated as well as their phenotype with flow cytometry. Further on, we determined their proliferative potential using population doublings (PD), PD time (PDT), and clonogenicity assay (CFU-F). Finally, we checked their genetic stability using RT-qPCR for TERT mRNA assay and karyotyping as well as their multilineage potential including adipogenic, chondrogenic, and osteogenic differentiation. Our results demonstrate that all the devices allow the production of SVF cells with consistent yield and viability, in less time than the reference method. Expanded cells from the four methods showed no significant differences in terms of phenotype, proliferation capabilities, differentiation abilities, and genetic stability.

## 1. Introduction

For many years, subcutaneous adipose tissue (AT) has been used for plastic and aesthetic surgery indications such as lipofilling and breast augmentation [1, 2]. However, the mechanism by which fat is able to stay at the site of injection was not fully understood. It is admitted that mesenchymal stem cells (MSCs) found in AT, that is, adipose-derived stromal cells (ASC) [3, 4], could be responsible for the engraftment success. Therefore, cell-assisted lipotransfer (CAL) has been developed to enrich the graft with SVF or ASC [5–7]. Nevertheless, this approach needs the isolation of the stromal vascular fraction (SVF) in the operating room, while the patient is under anesthesia. The reference method to isolate the SVF [4] is based on the series of washing, centrifugation, and collagenase digestion in a completely open system that is time-consuming and thus cannot be performed in operating room. Accordingly, companies have developed or are developing devices in order to provide an easy, fast, reliable, and closed system to isolate this SVF at the point of care [8–10]. These devices allow, on one hand, both the SVF extraction to be used to enrich the adipose tissue before grafting and, on the other hand, ASC isolation for expansion in GMP facilities for their use in the field of regenerative medicine.

ASC and all the MSCs share the same multilineage properties as their counterpart in the bone marrow [3, 4, 11, 12]. The Mesenchymal and Tissue Stem Cell Committee of the International Society for Cellular Therapy (ISCT) has proposed a minimal set of three criteria to define human MSCs [13] which have been recently updated [14]. Both SVF cells and cultured ASC have shown great promise in various therapeutic fields such as wound healing [15, 16], scleroderma [17, 18], osteoarthritis [19], erectile dysfunction [20], multiple sclerosis [21], and renal insufficiency [22] and numerous clinical trials are being conducted with the use of SVF or ASC (https://clinicaltrials.gov/).

The main goal of this study was to validate three devices allowing the isolation of the SVF for usage both in operating room for CAL purposes and in GMP facilities for advanced-therapy medicinal product (ATMP) development. In order to be confident to the current good manufacturing practices (cGMP) guidelines for the production of an ATMP, our criteria were the extraction yield, proliferation capabilities, multilineage potential, immunophenotype, and genetic stability. We therefore evaluated three medical devices: GID-SVF1 is already CE-labeled for SVF extraction; Puregraft is a fat bag allowing the washing and filtration of lipoaspirated fat and these inner properties were used in this study to isolate SVF; and Stem.pras is a device in development that we compared to the reference method regarding SVF cell yield and viability and phenotype, proliferation, multipotentiality, and genetic stability of cultured ASC.

## 2. Materials and Methods

### 2.1. Patients Selection

Surgical residue harvesting was performed according to French regulation including declaration to research ministry (DC number 2008162) and written information consent of the patients. The adipose tissue was harvested from the abdomen of 4 healthy donors undergoing an abdominal tumescent liposuction for cosmetic purpose. The mean age and BMI were 36.8 ± 5.13 (range 22–49) and 31.1 ± 2.41 (range 24.7–37.7) (mean ± SEM, *n* = 4), respectively.

### 2.2. Tissue Collection

For reference method and Puregraft, Adipose tissue was harvested via a 3 mm blunt cannula using a liposuction pump at −300 mmHg. Fat tissue was collected in a canister from where it was transferred to 60 cc syringes and then injected to the Puregraft filtration bag. For reference method, the canister was sent to the lab without additional manipulation within the operating room.

For Stem.pras and GID-SVF1, fat was harvested via a 3 mm blunt cannula using a liposuction pump at −300 mmHg in a closed system. The vacuum pump was connected into the device and the suction tube came from the device to the harvesting cannula. After suctioning, the fat was directly collected in the device without any manipulation for Stem.pras. For GID-SVF1, an additional filtration system allows the immediate separation of the fluid part from the collected fat.

### 2.3. Medical Devices Description (See Table 1 and Figure 1)

**Figure 1:**
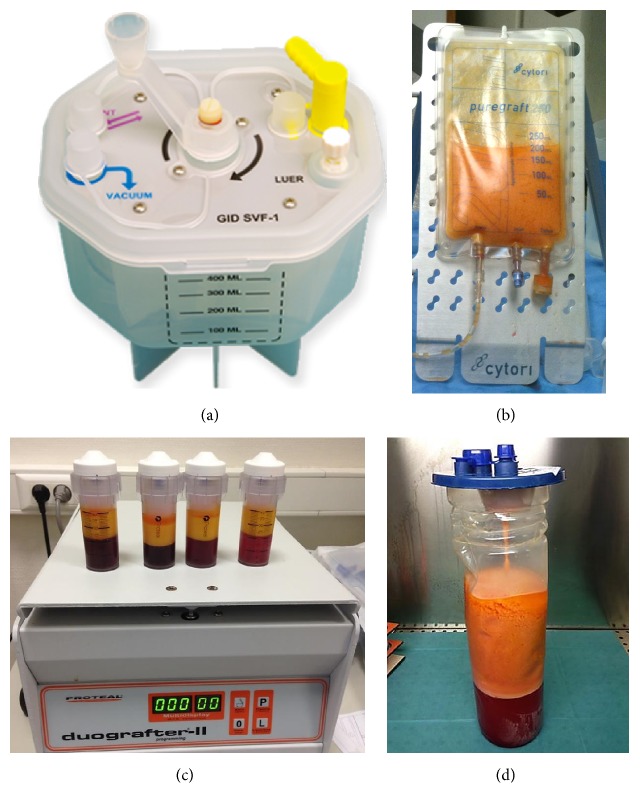
Devices used for comparative SVF isolation. (a) GID-SVF1. (b) Puregraft 250. (c) Stem.pras. (d) Reference method.

Available devices for SVF isolation were screened and three systems were selected according to several criteria (Table 1). The devices had to be convenient for its use in a surgical unit and GMP facilities and to provide viable cells with a yield not significantly different than reference method and provide genetically stable ASC cells meeting ISCT criteria:

1. *GID-SVF1* (Figure 1(a)) is from GID Europe. This CE-marked tool is designed to isolate the SVF cells from human lipoaspirate up to 350 g of dry adipose tissue (free of fluids). It enables the harvest of patient's lipoaspirate straight into the device, in a closed/safe sterile system, which allows direct filtration for separation of adipose tissue from its lipoaspirate fluids. It requires the use of GIDzyme-2 provided in the ready-to-use kit, a mix of collagenase optimized to dissociate the adipose tissue.
2. *Puregraft 250* (Figure 1(b)) is a purifying fat bag designed to wash and filter the fat before getting injected. Puregraft drains the tumescent fluid, free lipid, blood cells, and excess fluid from the graft [23]. In this study, we used its inner properties to design a new method to isolate SVF cells (see description below). Fat has to be harvested in an intermediate canister and then to be injected in the bag for filtration.
3. *Stem.pras* with Duografter II® (Figure 1(c)) from Proteal® provides a kit allowing the harvest of the fat and the isolation of SVF cells straight inside. It is designed as 50 cc canister (up to 4 canisters available in the kit) where fat can be harvested and then the canister is centrifuged. This device is not yet commercially available as we tested a prototype in development.

### 2.4. Lipoaspirate Processing

Each lipoaspirate from all patients was processed with all the devices.

The collagenase (NB6 GMP-grade, Serva Electrophoresis, Heidelberg, Germany) was used at a final concentration of 0.1 U/mL, except for GID-SVF1, as the manufacturer recommends the use of the GIDzyme-2.

Washing steps and digestion have been carried out using lactated Ringer (LR) solution (130 mmol/L Na^+^, 109 mmol/L Cl^−^, 28 mmol/L lactate, 1.5 mmol/L Ca^2+^, 4 mmol/L K^+^).

Tissue processing to obtain stromal vascular fraction was performed concurrently in all four systems.

#### 2.4.1. Reference Method

The SVF isolation was performed according to previously published method, with slight modifications [4]. Briefly, adipose tissue (Figure 1(d)) was washed 3 times in 20 mL syringes to remove lipoaspirate fluids, weighed, and then digested in LR (1 : 1, v/v, fat/LR) containing collagenase NB6 at a final concentration of 0.1 U/mL at 37°C for 45 min and under constant shaking. Digestion was stopped by adding Dulbecco's Modified Eagle's Medium (DMEM with GlutaMAX™, Gibco®, Carlsbad, USA) containing 10% fetal calf serum (FCS, HyClone, Thermo Fisher Scientific, Waltham, Massachusetts, USA). After centrifugation at 300*g*·min^−1^ for 5 min, floating adipocytes were discarded and cells from the SVF were pelleted, rinsed with media, centrifuged (300*g*·min^−1^ for 5 min, 20°C), and filtered through a 70 *μ*m sieve. Red blood cells (RBC) count was performed using a Pentra 60C+ (Horiba Medical, Kyoto, Japan). Cell pellet was then incubated in an erythrocyte lysis buffer (Hybri-Max™, Sigma-Aldrich, Saint-Quentin-Fallavier, France) for 20 min at 37°C. This cell suspension was centrifuged and total nucleated cells (TNC) were counted using Trypan blue exclusion method.

#### 2.4.2. Other Methods Using Medical Devices

For* GID-SVF1*, fat was collected in the device and isolation was performed as described previously [24]. After the third washing, the device was weighed to determine the total adipose tissue mass (GID-SVF1 tare was 254 g). For digestion, the fat was suspended in LR, to which an appropriate collagenase mix (GIDzyme-2 suspended in LR) was added. As recommended by the manufacturer, LR and the GIDzyme addition was adjusted to the amount of fat collected, with 1 vial for 100–175 g of fat. The device was incubated at 37°C for 45 minutes on a shaking rocker. The collagenase reaction was stopped by adding FCS in the device. The entire device was centrifuged for 10 minutes at 800*g*·min^−1^ and the oil layer removed. Using the manufacturer's protocol, the pellet was resuspended in LR and additional washes were performed after removal of the oil layer. After a second centrifugation, the remaining supernatant was removed and the pellet transferred to a 50 mL conical tube for the red blood cell numeration and the subsequent RBC lysis as described above.

For the* Puregraft 250*, fat was collected in an intermediate sterile canister and then transferred into the bag using a Toomey syringe. LR was injected into the bag using the “inlet” site to wash the lipoaspirate and contaminant fluids were removed using the “waste” site. After three washings, the bag was weighed to determine the total adipose tissue mass (Puregraft tare was 45 g). For digestion, the fat was suspended in LR (1 : 1, v/v, fat/LR), and collagenase NB6 was added at a final concentration of 0.1 U/mL. The device was incubated at 37°C for 45 minutes on a shaking rocker. The collagenase reaction was stopped by adding serum-containing medium. Then, tissue lysate was collected via the waste site, leaving remnant extracellular matrix in the bag, and transferred into 50 mL conical tube which was spun down at 300*g*·min^−1^ for 5 min. Floating adipocytes were discarded and cells from the SVF were pelleted, rinsed with media, centrifuged, 70 *μ*m sieve filtered, and incubated in an erythrocyte lysis buffer for 20 min at 37°C. This cell suspension was centrifuged and TNC were counted using Trypan blue exclusion method. Red blood cells count was performed using a Pentra 60C+.

For* Stem.pras*, fat was collected in four canisters as described above and LR was injected into each canister using the injection site to wash the lipoaspirate and contaminant fluids were removed using the same site. After three washings, the canisters were weighed to determine the total adipose tissue mass (canister tare was 45.5 g). For digestion, the fat was suspended in LR (1 : 1, v/v, fat/LR), and collagenase NB6 was added at a final concentration of 0.1 U/mL. The canisters were incubated at 37°C for 45 minutes on a shaking rocker. The collagenase reaction was stopped by adding serum-containing medium. Then, the canisters were spun down at 300*g*·min^−1^ for 5 min and floating adipocytes were suctioned with a syringe. Finally, cells from the SVF were collected, rinsed with media, centrifuged, and filtered through a 70 *μ*m sieve. RBC counting was performed as described above. Cell pellet was then incubated in an erythrocyte lysis buffer for 20 min at 37°C. This cell suspension was centrifuged and TNC were counted using Trypan blue exclusion method.

### 2.5. Evaluation Criteria

#### 2.5.1. Processing Time

Isolation was performed within two hours after tissue collection and fat was kept at room temperature until then. Processing time was approximately evaluated from the beginning of the isolation (first wash) to the obtaining of SVF (end of red blood cell lysis).

#### 2.5.2. SVF Cell Yield

SVF yield was calculated by dividing the number of viable TNC in SVF per gram of processed fat (i.e., dry fat weighed for collagenase digestion). Data are expressed as number of viable cells × 10^6^/g of dry fat ± standard error of the mean (SEM).

*Cultured ASC Characterization*

*(1) Phenotype*. SVF and the two subsequent subculture (named as P0 and P1) cells were characterized for their phenotype using flow cytometry approach. Briefly, after detachment, cells were resuspended in PBS at a concentration of 1-2 millions/mL. 100 *μ*L of this cell suspension was stained in PBS using FITC-coupled CD105, CD45, HLA-ABC and PE-coupled CD90, CD73, CD14, CD34, and HLA-DR antibodies (all from BD Biosciences, Le Pont de Claix, France). Fifty thousand events were acquired with a FACS Canto II (BD Biosciences) and analyzed (DIVA software) against appropriate isotypic controls.

*(2) Proliferation Capabilities and Clonogenic Potential (CFU-F Assay)*

  Proliferation was assessed as follows: freshly isolated SVF cells from the four techniques were plated at 40,000 cells/cm^2^ and cultured at 37°C in an atmosphere of 5% of carbon dioxide in humid air. Medium was replaced one hour after plating in order to remove nonadherent cells [23]. Proliferation medium was DMEM/F12 with GlutaMAX, 10% FCS, 100 U/mL penicillin, 100 *μ*g/mL gentamicin, 5 *μ*g/mL fungizone, and 10 ng/mL FGF2 premium grade (Miltenyi Biotec, Paris, France). Medium was replaced every 2-3 days and cells were detached at subconfluency using Trypsin-EDTA and replated at a density of 4,000 cell/cm^2^. The amount of population doubling (PD) was calculated as follows: PD = log⁡(*N*/*N*0)/log⁡2, where *N*0 is the seeded cell number and *N* the harvested cell number. The doubling time (DT) was calculated by dividing the PD by the number of hours between seeding time and detachment of the cells.
  Clonogenic potential was assessed as follows: cells recovered from the different devices were seeded at low density (40 cells/cm^2^) in triplicate in proliferation medium described above. Colonies were grown for 10 to 14 days, depending on the growth rate of the cells. At the end of the assay, the culture dishes were rinsed with phosphate-buffered saline twice and then fixed with 10% neutral buffered formalin for 20 minutes and then stained with 0.5% crystal violet (Sigma-Aldrich, Saint-Quentin-Fallavier, France). Cell colonies were counted using phase contrast microscopy. All three Petri dishes were counted, and the average and standard deviation were calculated to generate the final frequency percentage value expressed as colony forming efficiency (CFE) by dividing the number of colony counted by the number of cells seeded × 100.

*(3) Genetic Stability.* For karyotyping analysis, cells expanded at passage 1 were exposed for 3 hours to 0.7% colcemid (Life Technologies) diluted in the culture medium, and then cells were detached and centrifuged. The pellet was then resuspended in 0.075 mol/L KCl for 2 minutes at room temperature. Cells were centrifuged again, resuspended in methanol acetic acid (3 : 1) fixative, and stored at −20°C for at least 2 days. G-band staining was performed with the Leishman-Giemsa cocktail.

For TERT mRNA expression assay, RT-qPCR (see details below) was performed on cells expanded at passage 1. As positive control, MRC5-TERT cell line (cell line number 617, CelluloNet BioBank BB-0033-00072) transfected with Lenti-TERT virus (Cat number G200, ABM, Richmond, Canada) and the same untransfected cell line MRC5 (cell line number 494, CelluloNet BioBank BB-0033-00072) were used.

*(4) Multipotency*. For adipogenic differentiation, confluent cells at passage 1 were induced using adipogenic medium consisting of DMEM supplemented with 10% of FCS, 10 *μ*g/mL of 3-isobutyl-1-methylxanthine (IBMX), 100 *μ*M of indomethacin, 0.1 *μ*M of dexamethasone (All from Sigma-Aldrich, Saint-Quentin-Fallavier, France), and 200 mUI of insulin (Umulin, Lilly laboratories, Neuilly-sur-Seine, France). After 14 days, lipid droplets were stained using 0.4% Oil Red O solution after 10% formalin fixation, and total mRNA were harvested using RLT buffer and treated as described below (cf. RT-qPCR assay).

For osteogenic differentiation, subconfluent cells at passage 1 were induced using StemPro® Osteogenesis Differentiation Kit (Gibco®, Life Technologies, St Aubin, France). After 3 weeks, cells were fixed and stained with 40 mM Alizarin Red (Merck Millipore, Fontenay-sous-Bois, France) to visualize calcium deposition, and total mRNA were harvested using RLT buffer and treated as described below (cf. RT-qPCR assay).

Chondrogenic differentiation was evaluated using high-density pellet culture approach as previously described [25]. Briefly, subconfluent cells at P1 were detached using Trypsin-EDTA, numerated and 3.5 × 10^5^ viable ASCs were centrifuged (300*g*, 10 min, 2 times) in a V-bottom 96-well plate (BD Biosciences) to form a pellet. The pellets were treated for 21 days with defined chondrogenic medium, which consisted of DMEM-F12 (4.5 g/L glucose, Life Technologies, St Aubin, France), 1% of Insulin-Transferrin-Selenium (ITS, Life Technologies, St Aubin, France), 100 nM dexamethasone, 170 *μ*M L-ascorbic acid-2-phosphate, 1 mM sodium pyruvate, 350 *μ*M L-prolin, 10 ng/mL of transforming growth factor *β*3 (TGF*β*3, R&D Systems, Minneapolis, USA), and 50 ng/mL of bone morphogenetic protein (BMP2, Inductos, Medtronic Biopharma). On day 21, the pellet was harvested and fixed in 10% buffered formalin before embedding in paraffin. For histological analysis, tissue sections (3 *μ*m) were deparaffinized and rehydrated and chondrogenic differentiation was assessed by Alcian Blue staining of sulfated proteoglycans. Total mRNA were harvested from at least five pooled cell pellets using RLT buffer and treated as described below (cf. RT-qPCR assay).

### 2.6. RT-qPCR Assay

Tips and tubes for RT-qPCR were purchased from Sarstedt (Nümbrecht, Germany). Total RNA were extracted using RNeasy mini kit (Qiagen, Courtaboeuf, France) and assessed with NanoQuant system (Infinite® 200 Pro, Tecan, Männedorf, Suisse). cDNAs were first synthesized from 500 ng of total RNA in the presence of PrimeScript™ RT (PrimeScript RT Reagent Kit, Takara Biotechnology, Shiga, Japan), 25 pmol of Oligo dT primers, and 200 pmol of random hexamers. RT products were treated with 60 units of Ribonuclease H (Takara Biotechnology). The real-time PCR was performed using a RotorGene 6000 (Corbett Research, Mortlake, Australia) in a final volume of 20 *μ*L containing 5 *μ*L of a 60-fold dilution of the RT reaction medium, 15 *μ*L of reaction buffer from the Absolute qPCR SYBR® Green ROX Mix (Thermo Fischer Scientific), and 7.5 pmol of the specific forward and reverse primers (Sigma-Aldrich, Saint-Quentin-Fallavier, France). Primers were selected in order to span two different exons separated by an intron of at least 1 kbp so that genomic DNA contamination is of no concern (see Table 2).

Total amount of mRNA was normalized to endogenous GUSB mRNA that has been shown to be stable in culture and differentiation of ASC [26, 27]. Each assay was performed in duplicate and validation of the real-time PCR runs was assessed by evaluating the melting temperature of the products. mRNA relative quantification was done using 2^−ΔΔCt^ method. Results are presented as fold increase relative to control sample except for TERT quantification which was done using RT dilution as standard and results expressed as relative arbitrary units.

### 2.7. Practicability

Pros and cons for each method were noticed during the process.

### 2.8. Statistical Analysis

Data are presented as mean ± standard error of the mean (SEM). Each experiment has been performed to reach at least three repetitions (*n* ≥ 3). Differences were considered statistically significant by use of Kruskal-Wallis for multiple samples followed by Dunn's multiple comparisons test if they attained *p* < 0.05 (Prism, v4, GraphPad Software, Inc., La Jolla, CA, USA).

## 3. Results

### 3.1. Tissue Processing Time

Routine method allows the SVF isolation in more than 150 minutes, when Stem.pras process took about 110 minutes. Puregraft permitted the cell extraction in around 100 minutes and our GID-SVF1 allowed us to isolate SVF in approximatively 90 minutes.

### 3.2. SVF Cell Yield

**Figure 2:**
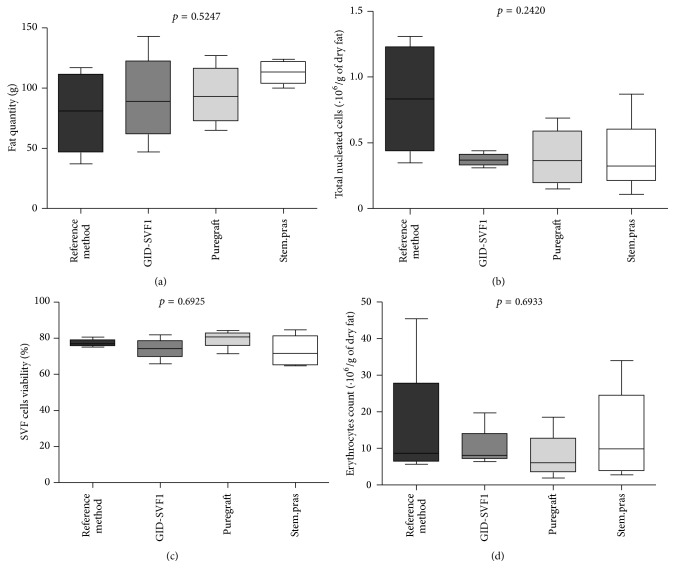
Fat quantity for the isolation, TNC count and their viability, and erythrocytes number remaining in the SVF. (a) Fat quantity (in grams) obtained after washing steps and therefore used for collagenase digestion. (b) Total nucleated cells obtained at the end of the process. (c) Viability of the isolated cells. (d) Remaining erythrocytes before the lysis step. The box contains the middle 50% of the data, with the upper edge indicating the 75th percentile of the data set and the lower edge indicating the 25th percentile. The line in the box indicates the median value of the data.

The quantity of fat harvested (Figure 2(a)), the total viable nucleated cells (Figure 2(b)), the viability of harvested cells (Figure 2(c)), and the remaining erythrocytes before the red blood cell lysis (Figure 2(d)) are shown in Figure 2. Globally, there are no significant differences regarding the parameters assessed. Indeed, the average quantity of dry adipose tissue obtained after the washing steps was 76 ± 15.53, 96.5 ± 18.68, 98.5 ± 17.56, and 107.25 ± 11.29 grams, respectively, for the reference method, GID-SVF1, Puregraft, and Stem.pras, respectively (Figure 2(a); *p* = 0.5247). No statistical difference was found in the yields of SVF cell isolation obtained from the four methods used in this study (Figure 2(b); *p* = 0.2420). Indeed, 0.795 ± 0.228, 0.425 ± 0.047, 0.25 ± 0.07, and 0.535 ± 0.209 × 10^6^ total nucleated cells per gram of dry fat were recovered from reference method, GID-SVF1, Puregraft, and Stem.pras, respectively. The mean viability of TNC was 75.8% ± 4.23, 81.47% ± 1.44, 77.45% ± 1.06, and 69.3% ± 2.41 when isolated using reference method, GID-SVF1, Puregraft, and Stem.pras, respectively (Figure 2(c), *p* = 0.6925). The number of remaining erythrocytes was determined before the red blood cell lysis buffer incubation and was 26.43 ± 8.3, 5.83 ± 1.23, 5.85 ± 1.75, and 11.88 ± 3.32 × 10^6^ per gram of dry fat when the SVF was obtained with reference method, GID-SVF1, Puregraft, and Stem.pras, respectively (Figure 2(d), *p* = 0.6933).

### 3.3. Cultured ASC Characteristics

#### 3.3.1. Phenotype

**Figure 3:**
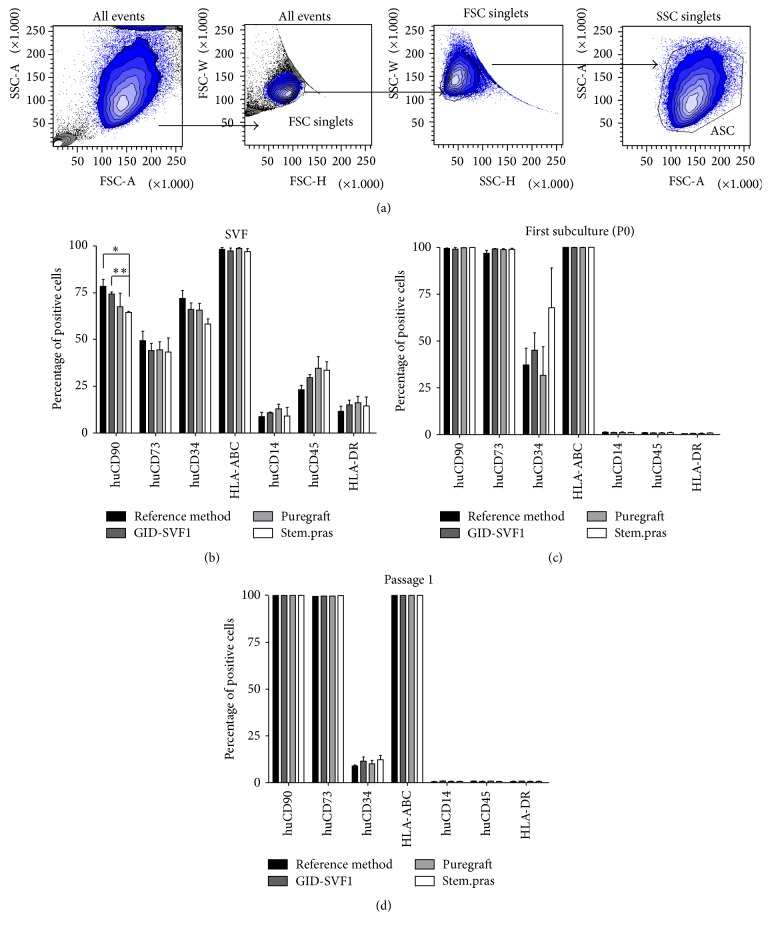
Detection of cell surface markers expressed by SVF cells and ASC at passages 0 and 1 by immunophenotyping and flow-cytometric analysis. (a) Gating strategy stream on morphological forward (FSC) and side scatter (SSC) used to eliminate debris and selected for cultured hASCs represented by contour plot. (b) Immunophenotyping of SVF cells. (c) Immunophenotyping of ASC at the first subculture (P0). (d) Immunophenotyping of ASC at the second subculture (P1). ^*∗*^*p* < 0.05; ^*∗∗*^*p* < 0.01.

SVF cells and P0 ASC and P1 ASC immunophenotypes were determined using flow cytometry and the results are displayed in Figure 3. The gating strategy was designed in order to exclude debris and doublets or aggregates (Figure 3(a)). For SVF analysis (Figure 3(b)), for all molecules screened, cells were not significantly different except for CD90 marker in Stem.pras isolated SVF cells when compared with GID-SVF1 and reference method but not with Puregraft (*p* < 0.01 and *p* < 0.05, resp.; *p* > 0.05 when compared with Puregraft). Classical CD73 mesenchymal marker and HLA-ABC were not statistically different between the devices (*p* > 0.05). In the same way, hematopoietic markers CD14, CD45, and HLA-DR were expressed at the same level in SVF (*p* > 0.05). Whatever the technique and medical device, expanded cells show the same phenotype as determined by flow cytometry. For P0 and P1 immunophenotype, the cells harbored a mesenchymal phenotype as shown in Figure 3(c) for P0 and Figure 3(d) for P1. Indeed, all the cells analyzed expressed CD90 and CD73 mesenchymal protein and HLA-ABC,and lacked the expression of CD14, CD45, and HLA-DR hematopoietic-related markers. Finally, cell expansion induced a dramatic decrease of CD34 expression from around 70% in the SVF to less than 10% on the surface of the cells at passage 1.

#### 3.3.2. Proliferation Potential

**Figure 4:**
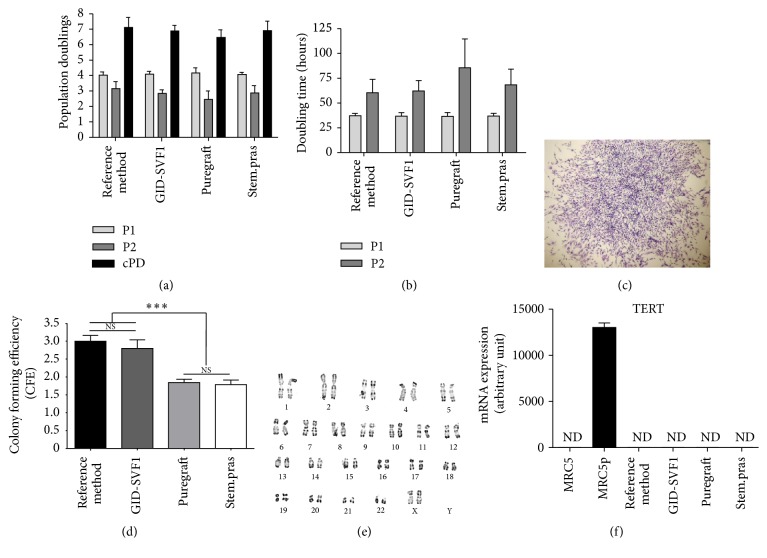
Growth kinetics and clonogenic potential of cells obtained with the four methods. (a) Population doubling (PD) at passages 1 and 2 and the corresponding cumulative PD for the four methods used to obtain the SVF. (b) Doubling time in hours for each subculture. (c) Representative photograph of a clone for CFU assay. (d) Colony forming efficiency calculated based on CFU assay. (e) All cell lines showed a normal karyotype with 23 chromosomes pairs and G-banding, indicating no chromosomal aberrations. (The karyotype shown is representative of the four methods evaluated.) (f) No hTERT expression was detected in the cell lines tested while positive control MRC5p hTERT-transfected cell line showed a high expression. CFE, colony forming efficiency; ND, not detected; NS, not significant. ^*∗∗∗*^*p* < 0.001.

Growth results of cultured cells from the four methods did not show any statistical differences as shown in Figure 4. Indeed, to compare the cell proliferation potential related to each medical device, cells were plated at 4,000 cells/cm^2^ and grown in proliferation medium for two subcultures. As shown in Figures 4(a) and 4(b), we did not find any statistical differences in the population doublings (PD), and thus cumulative PD and doubling times (DT) (see material and methods section for detailed calculation) were evaluated at the end of the culture. PD at the first (P1) and the second subculture (P2) were almost the same (*p* = 0.9363 and *p* = 0.8697, resp.) as the resulting cPD were *p* = 0.4753.

Moreover, to explore the clonogenic potential of SVF cells, we plated the cells at very low density (i.e., 40 cells/cm^2^), and evaluated their colony forming efficiency (CFE, Figure 4(c)). Reference method and GID-SVF1 methods allow isolating cells with the same apparent CFE (*p* > 0.05), as Puregraft and Stem.pras do (*p* > 0.05). However, reference method and GID-SVF1 were greater in obtaining clonogenic cells than Puregraft and Stem.pras (*p* < 0.001), as shown in Figure 4(d).

#### 3.3.3. Genetic Stability

None of the methods used in this study gave rise to genetically unstable cells, at least in regard to their karyotype (Figure 4(e)) and TERT expression (Figure 4(f)). Indeed, in order to check the possible genetic abnormalities of the ex vivo expanded cells, we carried out karyotyping analysis. No chromosomal modifications were observed in our culture condition, for every cells from all the devices used in this study (Figure 4(e)). We then also performed RT-qPCR experiments to verify the absence of expression of TERT mRNA (Figure 4(f)). Our results show the expression of TERT mRNA in MRC5-TERT cell line, while no expression was observed in untransfected MRC5 cell line and in our cells (Figure 4(f)).

#### 3.3.4. Multilineage Differentiation

**Figure 5:**
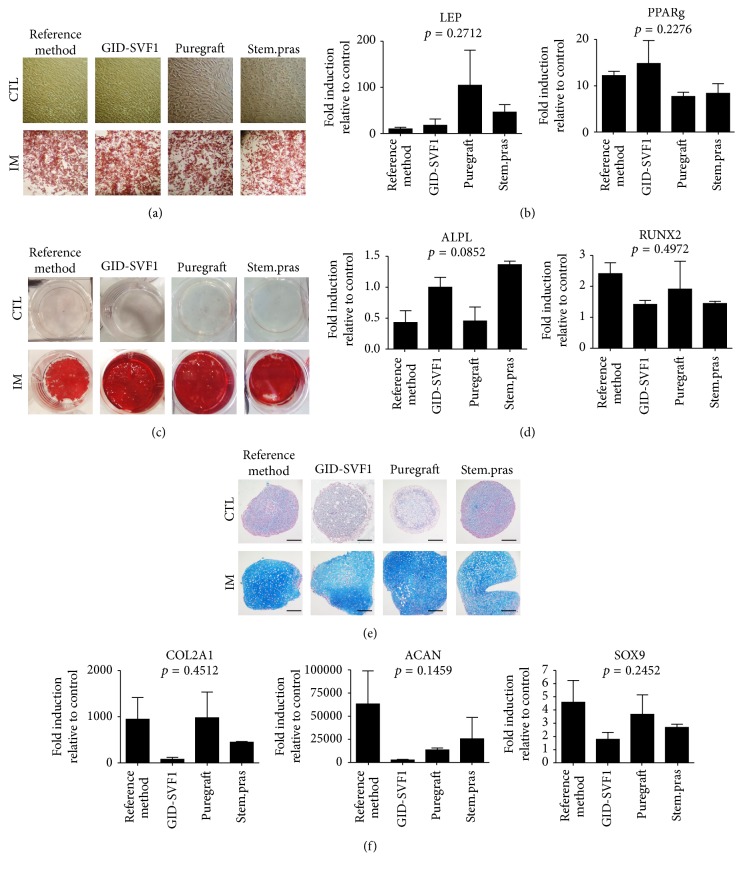
Multilineage differentiation of passage 1 adipose-derived stromal cell isolated using four different methods. (a) Cells either untreated (Ctl) or treated with adipogenic induction medium (IM) for 14 days and stained with Oil Red O. (b) RT-PCR assay for leptin and PPARG adipogenic specific genes. (c) Cells either untreated (Ctl) or treated with osteogenic induction medium (IM) for 21 days and stained with Alizarin Red. (d) RT-PCR assay for ALPL and RUNX2 osteogenic specific genes. (e) Cells either untreated (Ctl) or treated with chondrogenic induction medium (IM) for 21 days and stained with Alcian Blue. Scale bar 200 *μ*m. (f) RT-PCR assay for COL2A1, ACAN, and SOX9 chondrogenic specific genes.

To establish whether the cells obtained from the four methods were able to differentiate toward the three recommended lineages [11, 13], we exposed ASC at passage 1 to adipogenic, osteogenic, and chondrogenic medium. All the cell lines were able to differentiate to these mesodermal lineages (Figure 5). Oil Red O, Alizarin Red, and Alcian Blue staining were strongly positive using induction medium compared control condition for adipo-, osteo-, and chondrogenic differentiation, respectively. No significant differences were observed in specific genes for each differentiation assay (*p* > 0.05, Kruskal-Wallis).

### 3.4. Practicability of the Devices

All the devices evaluated allow an improvement of the process compared to the reference method. Indeed, GID SVF-1 and Stem.pras permit the harvest of lipoaspirated fat directly into the device. Puregraft was collected in an intermediate sterile container and then transferred into the bag using a Toomey syringe. Furthermore, GID-SVF1 and Stem.pras allow performing all the steps of the isolation process straight into the device, in a fully closed manner. GID-SVF1 is provided with its proprietary enzyme mixture, GIDzyme-2, as a ready-to-use device.

## 4. Discussion

The SVF and ASC bear great therapeutic potential in wide variety of reconstructive and regenerative applications [30–34]. Nevertheless, their use may be restricted by the challenges regarding their production as clinical grade products. Indeed, skilled staff, isolation duration, and logistic obstacle can hamper the viability, sterility, consistency, and functionality of the cell preparation. Mastering these hurdles is a hard goal to reach, and semiautomated devices for cell isolation are a crucial way to overcome these obstacles.

According to our defined criteria, the three evaluated devices, GID-SVF1 CE-labeled for SVF isolation, Puregraft firstly designed to wash and filter AT, and Stem.pras in current development for SVF isolation were validated for SVF extraction in operating room as well as for ASC culture in GMP facilities because no significant results were found versus the reference method. Indeed, regarding the SVF isolation, the devices are less time-consuming than reference method which take more than 150 minutes, whereas GID-SVF1, Puregraft, and Stem.pras need only 90, 100, and 110 minutes, respectively. These outcomes regarding process time are consistent with those found in the literature related to enzymatic digestion of lipoaspirated adipose tissue [8, 10, 35]. Furthermore, GID-SVF1 and Stem.pras allow the fat harvest, washing, and digestion in a closed manner, whereas reference method is a fully open process. In the case of Puregraft, it will be possible to harvest the lipoaspirated fat straight within the bag by using the Fat Lock™ system (commercially available from BE NEW Medical). However, this new system implies the fat suction with a syringe and cannot be used with a vacuum pump. Moreover, they permit reaching a cell yield upper than 2.5 × 10^5^ cells/gram of dry AT (from 2.5 × 10^5^ ± 0.07 to 7.95 × 10^5^ ± 0.21) with viability greater than 69% (from 69.3 ± 2.4 to 81.46 ± 1.4) which is also consistent with already published data [8, 35, 36]. Finally, SVF cells show a high CD34 and CD90 and a weak CD45 proportion, a frequency of CFU-F upper than 1% and with a trilineage potential. Altogether, our results fulfill the recommendations advised by the ISCT and the International Federation for Adipose Therapeutics (IFATS) [14]. Regarding grown cells obtained from the devices from SVF plating in culture dishes, no karyotype abnormalities and no hTERT expression were shown, thus highlighting a genetic stability.

Furthermore, duration of collagenase digestion has been correlated with poor cell viability [37]. In our design, we set the time of enzymatic treatment to be the same as our reference technique validated in our GMP process in order to reach the best compromise for both cell yield and viability. Regarding collagenase, we set the concentration at 0.1 U/mL (GMP) to standardize and limit the variability of our process, except for GID-SVF1 in which collagenase is provided in a ready-to-use kit with unknown concentration. The digestion time and concentration of collagenase have been determined previously in our lab (unpublished data). Despite this restriction, our results were not significantly different in terms of the cell yield and viability. The great benefit of these systems when compared with reference method is that they allow (or with slight modification) the harvest of SVF cells in a closed system from the patients to the patient or from patient to culture dishes easily.

Those devices facilitate washing of lipoaspirated fat. Washing steps are crucial to reduce undesirable components, such as blood, debris, free lipid, and ruptured adipocytes, while retaining viable adipose tissue for further use. The most common release criteria value for cell viability is upper 70% [14]. On average, all the devices tested in this study meet this crucial criterion. However, two of the values only for Stem.pras were under 70%. In our hands, the four methods are similar regarding their washing efficacy since we did not show any statistical difference in the remaining erythrocytes in the SVF. The presence of red blood cells in the SVF in the context of its use for ASC expansion is not a major issue since these contaminants are not adherents and are removed from the culture dish by the medium change after one hour in our protocol.

Regarding the immunophenotype, whatever the devices used, more than 64% and more than 43% of the isolated cells expressed mesenchymal markers CD90 and CD73, respectively, and more than 58% expressed putative ASC CD34 molecule [14, 38]. However, it would be of great interest to perform multiparametric flow cytometry in order to highlight the exact proportion of native ASC in the SVF as they are defined by the expression of CD34 and the associated lack of CD31 endothelial marker and CD45 pan-leukocyte markers. Furthermore, CD34 native ASC phenotype has been shown to disappear during cell expansion, an observation that we confirm in our results. In our point of view, monochromatic flow cytometry should be considered as sufficient for the screening of classical markers of ASC since these molecules have an “ON/OFF” expression pattern on cultured ASC from two or three subcultures [39, 40]. Moreover, our results show that our grown cells, whatever the device, harbor the mesenchymal recommended phenotype [13].

In vitro and under specific stimuli, ASCs have been shown to have a wide multipotency [41]. Here we show that our cells are able to differentiate toward adipogenic, osteogenic, and chondrogenic lineages with no significant differences regarding gene specific expression. In a previous study from our team, we demonstrate the ability of ASC to engage toward endothelial lineage [12].

In the context of ATMP manufacturing after cell expansion, it is crucial to verify the genetic stability [42–44]. Our results did not show any genetic instability using karyotyping and TERT mRNA was not detectable as determined by RT-qPCR approach. Transformation process is a complicated sequence of events which might occur when the cells are grown in vitro [45, 46]. Nowadays, there is no evidence of a real genetic instability of ASC and MSC in general [47], but a small number of publications highlighted few aberrations [48, 49], and some publications [50, 51] have been retracted due to cell culture contamination [52, 53]. Moreover, we deeply think that closed system that strikingly limit the microbial contamination allow surgical unit and GMP-laboratories to preserve the safety of the patient.

Regarding the quality controls and the conformity to the regulation, all methods described in this study match the recommendations. Consequently, all of them can be used and chosen depending on their availability in the country or their cost, for example. Regarding the practicability, GID-SVF1 appears to be the most appropriate device for a surgeon in an operating room as it is provided as a ready-to-use kit including the collagenase. A new version of this device, the GID-SVF2™ has recently been compared to other devices [35]. Even if it has to be confirmed, it appears that this new version is smaller and yield less TNC and CFU than the GID-SVF1 tested in this study. From the same point of view, Proteal could improve its Stem.pras system simply by adding a ready-to-use collagenase. Puregraft is already used in operating room for autologous fat transfer [54] and, thanks to our results, surgeons could use this tool to perform CAL. Similarly, Puregraft could be improved by adding a ready-to-use collagenase. Finally, GID-SVF1, Stem.pras, and Puregraft system are suitable for cell therapy labs equipped with GMP facilities.

Nevertheless, it is possible to obtain ASC without the use of any device, as published for the explanted lipoaspirate or adipose tissue [55]. These approaches allow efficiently obtaining ASC, in a more economic and faster manner, but the techniques we present in this paper bring the close processing of the tissue. Even if we did not test the sterility of the SVF obtained from each devices, this aspect is of great interest because it maximizes the probability to obtain a sterile SVF. Nevertheless, none of the SVF seeded showed any microbiological development in vitro. Lastly, a number of papers report the absence of residual collagenase activity in their final products [8, 35]. In 2013, it has been reported that there was no detectable residual collagenase activity after the first wash. In our study, we use a low collagenase concentration (0.1 U/mL), and we performed 2 washing steps which altogether allow presumably the lowering of the residual collagenase activity to an undetectable level.

The SVF isolation devices market is expanding for many years, and it is obviously important to check their efficacy before the transfer from bench to bedside.

Our results clearly demonstrate that the three evaluated closed devices, GID-SVF1, Puregraft, and Stem.pras, are equivalent to reference method in terms of SVF cells yield, viability, phenotype, and clonogenic potential. We also found that their expanded cells are similar in terms of phenotype, proliferation potential, multipotency, and genetic stability.

## Figures and Tables

**Table 1:** List of devices for SVF isolation.

Device	Company	Open/ semiclosed^*∗*^/ closed	Automated/ semiautomated/ manual	Maximum fat quantity (g)	Method for fluid separation	Collagenase provided (yes/no)	Disposable cost ($)	Fat treatment duration (minutes)	Publication
Celution® 800/CRS	Cytori Therapeutics, Inc. http://www.cytori.com	Semiclosed	Automated	300	Decantation	Yes (Celase™)	1950	90	[8]

Cha-Station™	PNC International Co., Ltd. http://inphronics.com.sg/equipment/cha-station	Semiclosed	Semiautomated	200	Not provided	No	710	90	[8]

GID SVF-1	GID Group, Inc. http://www.thegidgroup.com/	Closed	Manual	300	Filtration	Yes (GIDzyme-2)	500	90	[24, 28]

Lipokit with Maxstem	Medi-Khan http://www.medikanint.com/	Semiclosed	Manual	100	Centrifugation	No	530	110	[8]

Multi Station	PNC International Co., Ltd. PNC North America http://pncint.business1.com/	open	Manual	100–150	Centrifugation	No	460	110	[8]

Puregraft 250	Eurosilicone	Semiclosed	Manual	250	Filtration	No	250	100^†^	[23]

Sepax®	Biosafe Group SA https://www.biosafe.ch	Semiclosed	Semiautomated	300	Not provided	No	420	90–120	[29]

Stem.pras with Duografter II	Proteal	closed	Manual	200^†^	Decantation	No	Unknown	110^†^	None

Tissue Genesis® Icellator	Tissue Genesis, Inc. http://www.tissuegenesis.com/icellator	Semiclosed	Semiautomated	60	Not provided	Yes Adipase®	Unknown	80	[9]

^†^Information added from this study

^*∗*^Semiclosed relies to those that do not allow the lipoaspirate to be harvested direct in the device.

**Table 2:** List of primers used for RT-qPCR experiments.

Gene name	Alternative name	Accession numbers	Forward 5′-3′	Reverse 5′-3′	Size (bp)
GUSB		NM_000181	TGGTTGGAGAGCTCATTTGG	CTCTCGCAAAAGGAACGCTG	131

TERT		NM_001193376 NM_198253	GCACCAACATCTACAAGATCC	GACATCCCTGCGTTCTTGGC	181

LEP	OB	NM_008493	CACCAGGATCAATGACATTTC	TGCCAGTGTCTGGTCCATCTTG	124

PPARG		NM_138711 NM_015869 NM_005037 NM_138712	TCTCTCCGTAATGGAAGACC	GCATTATGAGACATCCCCAC	474

RUNX2		NM_001015051 NM_001024630 NM_001278478	ACCAGATGGGACTGTGGTTAC	AGACGGTTATGGTCAAGGTG	167

ALPL	AP	NM_000478 NM_001127501 NM_001177520	GACCTCGTTGACACCTGGAAG	TTCCTGTTCAGCTCGTACTGC	155

SOX9		NM_000346.3	CCCAACGCCATCTTCAAGG	GGTCAAACTCGTTGACATCG	289

COL2A1		NM_001844 NM_033150	TCCATGTTGCAGAAAACCTTCA	GGAAGAGTGGAGACTACTGGATTGAC	76

ACAN	AGC1	NM_001135 NM_013227	TCGAGGACAGCGAGGCC	TCGAGGGTGTAGCGTGTAGAGA	85

## References

[1] Smahel J. (1986). Adipose tissue in plastic surgery. *Annals of Plastic Surgery*.

[2] Mojallal A., Foyatier J.-L. (2004). Historical review of the use of adipose tissue transfer in plastic and reconstructive surgery. *Annales de Chirurgie Plastique et Esthétique*.

[3] Zuk P. A., Zhu M., Ashjian P. (2002). Human adipose tissue is a source of multipotent stem cells. *Molecular Biology of the Cell*.

[4] Zuk P. A., Zhu M., Mizuno H. (2001). Multilineage cells from human adipose tissue: implications for cell-based therapies. *Tissue Engineering*.

[5] Matsumoto D., Sato K., Gonda K. (2006). Cell-assisted lipotransfer: supportive use of human adipose-derived cells for soft tissue augmentation with lipoinjection. *Tissue Engineering*.

[6] Sterodimas A., de Faria J., Nicaretta B., Papadopoulos O., Papalambros E., Illouz Y. G. (2010). Cell-assisted lipotransfer. *Aesthetic Surgery Journal*.

[7] Yoshimura K., Sato K., Aoi N., Kurita M., Hirohi T., Harii K. (2008). Cell-assisted lipotransfer for cosmetic breast augmentation: supportive use of adipose-derived stem/stromal cells. *Aesthetic Plastic Surgery*.

[8] Aronowitz J. A., Ellenhorn J. D. I. (2013). Adipose stromal vascular fraction isolation: a head-to-head comparison of four commercial cell separation systems. *Plastic and Reconstructive Surgery*.

[9] Doi K., Tanaka S., Iida H. (2013). Stromal vascular fraction isolated from lipo-aspirates using an automated processing system: bench and bed analysis: SVF from lipoaspirates using automated processing. *Journal of Tissue Engineering and Regenerative Medicine*.

[10] Raposio E., Caruana G., Petrella M., Bonomini S., Grieco M. P. (2016). A standardized method of isolating adipose-derived stem cells for clinical applications. *Annals of Plastic Surgery*.

[11] Pittenger M. F. (1999). Multilineage potential of adult human mesenchymal stem cells. *Science*.

[12] Auxenfans C., Lequeux C., Perrusel E., Mojallal A., Kinikoglu B., Damour O. (2012). Adipose-derived stem cells (ASCs) as a source of endothelial cells in the reconstruction of endothelialized skin equivalents. *Journal of Tissue Engineering and Regenerative Medicine*.

[13] Dominici M., Le Blanc K., Mueller I. (2006). Minimal criteria for defining multipotent mesenchymal stromal cells. The International Society for Cellular Therapy position statement. *Cytotherapy*.

[14] Bourin P., Bunnell B. A., Casteilla L. (2013). Stromal cells from the adipose tissue-derived stromal vascular fraction and culture expanded adipose tissue-derived stromal/stem cells: a joint statement of the International Federation for Adipose Therapeutics and Science (IFATS) and the International Society for Cellular Therapy (ISCT). *Cytotherapy*.

[15] Hassan W. U., Greiser U., Wang W. (2014). Role of adipose-derived stem cells in wound healing. *Wound Repair and Regeneration*.

[16] Rodriguez J., Boucher F., Lequeux C. (2015). Intradermal injection of human adipose-derived stem cells accelerates skin wound healing in nude mice. *Stem Cell Research and Therapy*.

[17] Granel B., Daumas A., Jouve E. (2015). Safety, tolerability and potential efficacy of injection of autologous adipose-derived stromal vascular fraction in the fingers of patients with systemic sclerosis: an open-label phase I trial. *Annals of the Rheumatic Diseases*.

[18] Guillaume-Jugnot P., Daumas A., Magalon J. (2016). Autologous adipose-derived stromal vascular fraction in patients with systemic sclerosis: 12-month follow-up. *Rheumatology*.

[19] Ruiz M., Cosenza S., Maumus M., Jorgensen C., Noël D. (2016). Therapeutic application of mesenchymal stem cells in osteoarthritis. *Expert Opinion on Biological Therapy*.

[20] You D., Jang M. J., Kim B. H. (2015). Comparative study of autologous stromal vascular fraction and adipose-derived stem cells for erectile function recovery in a rat model of cavernous nerve injury. *Stem Cells Translational Medicine*.

[21] Riordan N. H., Ichim T. E., Min W.-P. (2009). Non-expanded adipose stromal vascular fraction cell therapy for multiple sclerosis. *Journal of Translational Medicine*.

[22] Matillon X., Baulier E., Hauet T. (2015). Characterization of mesenchymal stem cells from pig fat and their effect on delayed graft function of transplanted kidney in a preclinical pig model of auto-transplantation mimicking the non-heart-beating donors conditions. *Progres en Urologie*.

[23] Zhu M., Cohen S. R., Hicok K. C. (2013). Comparison of three different fat graft preparation methods: gravity separation, centrifugation, and simultaneous washing with filtration in a closed system. *Plastic and Reconstructive Surgery*.

[24] Dos-Anjos Vilaboa S., Navarro-Palou M., Llull R. (2014). Age influence on stromal vascular fraction cell yield obtained from human lipoaspirates. *Cytotherapy*.

[25] Johnstone B., Hering T. M., Caplan A. I., Goldberg V. M., Yoo J. U. (1998). In vitro chondrogenesis of bone marrow-derived mesenchymal progenitor cells. *Experimental Cell Research*.

[26] Fink T., Lund P., Pilgaard L., Rasmussen J. G., Duroux M., Zachar V. (2008). Instability of standard PCR reference genes in adipose-derived stem cells during propagation, differentiation and hypoxic exposure. *BMC Molecular Biology*.

[27] Tratwal J., Follin B., Ekblond A., Kastrup J., Haack-Sørensen M. (2014). Identification of a common reference gene pair for qPCR in human mesenchymal stromal cells from different tissue sources treated with VEGF. *BMC Molecular Biology*.

[28] Rose L. C., Kadayakkara D. K., Wang G. (2015). Fluorine-19 labeling of stromal vascular fraction cells for clinical imaging applications. *Stem Cells Translational Medicine*.

[29] Güven S., Karagianni M., Schwalbe M. (2012). Validation of an automated procedure to isolate human adipose tissue-derived cells by using the sepax® technology. *Tissue Engineering Part C: Methods*.

[30] Brown S. A., Levi B., Lequex C., Wong V. W., Mojallal A., Longaker M. T. (2010). Basic science review on adipose tissue for clinicians. *Plastic and Reconstructive Surgery*.

[31] Mendicino M., Bailey A. M., Wonnacott K., Puri R. K., Bauer S. R. (2014). MSC-based product characterization for clinical trials: an FDA perspective. *Cell Stem Cell*.

[32] Nordberg R. C., Loboa E. G. (2015). Our fat future: translating adipose stem cell therapy. *Stem Cells Translational Medicine*.

[33] Salem H. K., Thiemermann C. (2010). Mesenchymal stromal cells: current understanding and clinical status. *STEM CELLS*.

[34] Tremp M., Salemi S., Gobet R., Sulser T., Eberli D. (2011). Adipose-Derived Stem Cells (ASCs) for tissue engineering. *Regenerative Medicine and Tissue Engineering—Cells and Biomaterials*.

[35] Aronowitz J. A., Lockhart R. A., Hakakian C. S., Birnbaum Z. E. (2016). Adipose stromal vascular fraction isolation: a head-to-head comparison of 4 cell separation systems #2. *Annals of Plastic Surgery*.

[36] Markarian C. F., Frey G. Z., Silveira M. D. (2014). Isolation of adipose-derived stem cells: a comparison among different methods. *Biotechnology Letters*.

[37] Seaman S. A., Tannan S. C., Cao Y., Peirce S. M., Lin K. Y. (2015). Differential effects of processing time and duration of collagenase digestion on human and murine fat grafts. *Plastic and Reconstructive Surgery*.

[38] Maumus M., Peyrafitte J.-A., D'Angelo R. (2011). Native human adipose stromal cells: localization, morphology and phenotype. *International Journal of Obesity*.

[39] Pachón-Peña G., Yu G., Tucker A. (2011). Stromal stem cells from adipose tissue and bone marrow of age-matched female donors display distinct immunophenotypic profiles. *Journal of Cellular Physiology*.

[40] Yoshimura K., Shigeura T., Matsumoto D. (2006). Characterization of freshly isolated and cultured cells derived from the fatty and fluid portions of liposuction aspirates. *Journal of Cellular Physiology*.

[41] Gimble J. M., Guilak F. (2003). Differentiation potential of Adipose Derived Adult Stem (ADAS) cells. *Current Topics in Developmental Biology*.

[42] Goldring C. E. P., Duffy P. A., Benvenisty N. (2011). Assessing the safety of stem cell therapeutics. *Cell Stem Cell*.

[43] Heslop J. A., Hammond T. G., Santeramo I. (2015). Concise review: workshop review: understanding and assessing the risks of stem cell-based therapies. *Stem Cells Translational Medicine*.

[44] Sensebé L. (2013). Beyond genetic stability of mesenchymal stromal cells. *Cytotherapy*.

[45] Chow M., Rubin H. (2000). Clonal selection versus genetic instability as the driving force in neoplastic transformation. *Cancer Research*.

[46] Hayflick L., Moorhead P. S. (1961). The serial cultivation of human diploid cell strains. *Experimental Cell Research*.

[47] Sharma S., Bhonde R. (2015). Mesenchymal stromal cells are genetically stable under a hostile in vivo-like scenario as revealed by in vitro micronucleus test. *Cytotherapy*.

[48] Bigot N., Mouche A., Preti M. (2015). Hypoxia differentially modulates the genomic stability of clinical-grade ADSCs and BM-MSCs in long-term culture. *STEM CELLS*.

[49] Buyanovskaya O. A., Kuleshov N. P., Nikitina V. A., Voronina E. S., Katosova L. D., Bochkov N. P. (2009). Spontaneous aneuploidy and clone formation in adipose tissue stem cells during different periods of culturing. *Bulletin of Experimental Biology and Medicine*.

[50] Røsland G. V., Svendsen A., Torsvik A. (2009). Long-term cultures of bone marrow-derived human mesenchymal stem cells frequently undergo spontaneous malignant transformation. *Cancer Research*.

[51] Rubio D., Garcia-Castro J., Martín M. C. (2005). Spontaneous human adult stem cell transformation. *Cancer Research*.

[52] Garcia S., Martín M. C., de la Fuente R., Cigudosa J. C., Garcia-Castro J., Bernad A. (2010). Pitfalls in spontaneous in vitro transformation of human mesenchymal stem cells. *Experimental Cell Research*.

[53] Torsvik A., Røsland G. V., Svendsen A. (2010). Spontaneous malignant transformation of human mesenchymal stem cells reflects cross-contamination: putting the research field on track—letter. *Cancer Research*.

[54] Mestak O., Sukop A., Hsueh Y.-S. (2014). Centrifugation versus PureGraft for fatgrafting to the breast after breast-conserving therapy. *World Journal of Surgical Oncology*.

[55] Priya N., Sarcar S., Majumdar A. S., Sundarraj S. (2014). Explant culture: a simple, reproducible, efficient and economic technique for isolation of mesenchymal stromal cells from human adipose tissue and lipoaspirate. *Journal of Tissue Engineering and Regenerative Medicine*.

